# The Ameliorating Effect of Myrrh on Scopolamine-Induced Memory Impairments in Mice

**DOI:** 10.1155/2015/925432

**Published:** 2015-11-09

**Authors:** Samrat Baral, Du-Hyong Cho, Ramesh Pariyar, Chi-Su Yoon, Bo-yoon Chang, Dae-Sung Kim, Hyoung-Kwon Cho, Sung Yeon Kim, Hyuncheol Oh, Youn-Chul Kim, Jaehyo Kim, Jungwon Seo

**Affiliations:** ^1^Institute of Pharmaceutical Research and Development, College of Pharmacy, Wonkwang University, Iksan 570-749, Republic of Korea; ^2^Hanbang Body-Fluid Research Center, Wonkwang University, Iksan 570-749, Republic of Korea; ^3^Department of Pharmacology, School of Medicine, Eulji University, Jung-gu, Daejeon 301-746, Republic of Korea; ^4^Standardized Material Bank for New Botanical Drugs, College of Pharmacy, Wonkwang University, Iksan 570-749, Republic of Korea; ^5^Hanpoong Pharm & Foods Co., Ltd., Jeonju 561-841, Republic of Korea; ^6^Department of Meridian & Acupoint, College of Korean Medicine, Wonkwang University, Iksan 570-749, Republic of Korea

## Abstract

Myrrh has been used since ancient times for the treatment of various diseases such as inflammatory diseases, gynecological diseases, and hemiplegia. In the present study, we investigated the effects of aqueous extracts of myrrh resin (AEM) on scopolamine-induced memory impairments in mice. AEM was estimated with (2*E*,5*E*)-6-hydroxy-2,6-dimethylhepta-2,4-dienal as a representative constituent by HPLC. The oral administration of AEM for 7 days significantly reversed scopolamine-induced reduction of spontaneous alternation in the Y-maze test. In the passive avoidance task, AEM also restored the decreased latency time of the retention trial by scopolamine treatment. In addition, Western blot analysis and Immunohistochemistry revealed that AEM reversed scopolamine-decreased phosphorylation of Akt and extracellular signal-regulated kinase (ERK). Our study demonstrates for the first time that AEM ameliorates the scopolamine-induced memory impairments in mice and increases the phosphorylation of Akt and ERK in the hippocampus of mice brain. These results suggest that AEM has the therapeutic potential in memory impairments.

## 1. Introduction

Memory impairment can be caused not only by aging or stress, but also by the neurodegenerative diseases such as Alzheimer's disease (AD). The loss of cholinergic function by cholinergic neuronal degeneration in the central nervous system significantly contributes to the cognitive decline associated with AD [1]. Accordingly, scopolamine, a competitive antagonist for muscarinic acetylcholine receptor (mAChR), induces memory impairments in rodents which parallel those in AD patients [2]. It is assumed that the scopolamine-induced amnesic animal model is very useful tool for screening the protective agents against memory impairment of AD symptoms [3, 4]. Acetylcholinesterase (AChE) is a key enzyme for hydrolysis of acetylcholine; thereby it regulates cholinergic function. Indeed, AChE inhibitors such as donepezil and rivastigmine are prescribed for ameliorating AD symptoms [5]. However, these medicines for AD treatment have side effects: hepatotoxicity, nausea, and diarrhea are concerns [4, 6]. Therefore, the interest has been drawn towards developing natural product based drugs which are generally more accessible with few or no side effects [7, 8].

One of the molecular signaling pathways associated with memory functions is extracellular signal-regulated kinase (ERK). ERK1/2, a member of mitogen-activated protein kinase superfamily, is expressed ubiquitously, conserved well, and responsible for intracellular response transmitted from extracellular signal. The activation of mAChRs in the neurons induces the elevation of intracellular calcium level, phosphoinositol turnover that activates ERK [9]. ERK activation is necessary for the establishment of long-term potentiation (LTP), the cellular mechanism underlying synaptic plasticity and memory [10]. In addition, Akt is another signaling molecule involved in learning and memory. Akt activation is also necessary for hippocampal LTP induction [11] and the inhibition of Akt induces memory impairments in passive avoidance task [12] and radial arm maze task [13]. Accumulating researches have shown that scopolamine decreases the phosphorylation of both Akt and ERK in the brain of scopolamine-treated mice [14, 15].

Myrrh*, Commiphora myrrha* or* Commiphora molmol* Engler, belongs to the family Burseraceae. It is found in abundance in the dry and arid regions of Ethiopia, Somalia, and Northern Kenya [16] and also habitat in some Asian countries [17]. It exists as a large shrub or a small tree which yields a yellow nonvolatile gum resin. It has been used since ancient times for the treatment of inflammatory diseases, gynecological diseases, wounds, pain, obesity, and hemiplegia [18]. Myrrh is heavily composed of water-soluble gum (30–60%), alcohol-soluble resins (25–40%), and small proportion of essential oil (3–8%) [19]. The characteristic constituents of myrrh oil include furanosesquiterpenes such as furanoelemanes, furanoeudesmanes, and furanogermacrenes. Previous studies demonstrated that the extracts of Myrrh had anti-inflammatory and analgesic effects [20]. The ethanol, petroleum ether, or water extracts of myrrh reduced acetic acid-induced writhing response and formalin-induced paw swelling along with the decreased levels of inflammatory factor prostaglandin E_2_ (PGE_2_) in mice. It has also been reported that sesquiterpenes isolated from the resins of myrrh showed neuroprotective effects against MPP^+^ induced neuronal cell death in SH-SY5Y cells [21]. However, the molecular mechanism of its neuroprotective effects entirely remains to be elucidated. In addition, the effect of myrrh on memory has not been reported yet. Therefore, we tested whether the aqueous extracts of myrrh resin (AEM) ameliorated memory impairments and found that oral administration of AEM improved scopolamine-induced memory impairments using passive avoidance task and Y-maze test. Furthermore, AEM reversed scopolamine-decreased phosphorylation of Akt and ERK in mice hippocampus, suggesting the potential role of Akt and ERK in AEM-improved memory impairments.

## 2. Materials and Methods

### 2.1. Chemical Material

(−)-Scopolamine hydrobromide (scopolamine) and 9-Amino-1,2,3,4-tetrahydroacridine hydrochloride hydrate (tacrine) were purchased from Sigma. Scopolamine and tacrine were dissolved in 0.9% saline solution for animal administration.

### 2.2. Extract Preparation

The resin of myrrh was purchased from Dong Kyung Pharm. Co. located at 128, Yangnyeongdong-gil, Dongdaemun-gu, Seoul, Republic of Korea. This resin was authenticated by Professor Ju at the School of Korean Medicine, Woosuk University, Samrye, Jeonbuk, Republic of Korea. A voucher specimen (HP-2014-12) of this material was deposited in the herbarium of Hanpoong Pharm & Foods Co. Ltd., Jeonju, Republic of Korea. The resin of myrrh (500 g) was extracted with hot water (10 L) for 3 h and filtrate was evaporated under reduced pressure to give residues (AEM; 188 g; 37.6 w/w%). AEM was prepared in 0.9% saline solution for animal administration.

### 2.3. Extraction and Isolation of (2*E*,5*E*)-6-Hydroxy-2,6-dimethylhepta-2,4-dienal

AEM (50 g) was suspended in H_2_O (1 L) and partitioned with EtOAc (1.5 L) to give EtOAc (MYE) and aqueous fraction (MYW). The MYE fraction was fractionated using a silica gel column chromatography, eluted with hexane in EtOAc (3 : 1–1 : 1, stepwise), and 50% chloroform in methanol to provide five subfractions (MYE1–5). The MYE2 subfraction was subjected to a reversed phase (RP) C_18_ column chromatography, eluted with methanol (40%–70%, stepwise) in water to provide (2*E*,5*E*)-6-hydroxy-2,6-dimethylhepta-2,4-dienal. The structure of this compound was identified from the analysis of NMR data with a comparison of its spectral data to those reported in the literature [22]. NMR spectra were recorded in CD_3_OD with a JEOL JNM ECP-400 spectrometer, and the chemical shifts were referenced relative to the residual solvent peaks (*δ*_H_/*δ*_C_ = 3.30/49.0).

(2*E*,5*E*)-6-Hydroxy-2,6-dimethylhepta-2,4-dienal: ^1^H NMR data (400 MHz, CD_3_OD) *δ*: 1.34 (6H, s, H-7, H-9), 1.82 (3H, d, *J* = 1.2 Hz, H-8), 6.42 (1H, d, *J* = 15.6 Hz, H-5), 6.78 (1H, dd, *J* = 15.2, 11.2 Hz, H-4), 7.01 (1H, d, *J* = 11.2 Hz, H-3), 9.40 (1H, s, H-1). ^13^C NMR data (100 MHz, CD_3_OD) *δ*: 8.8 (C-8), 28.3 (C-7, C-9), 70.3 (C-6), 121.4 (C-4), 137.2 (C-2), 149.3 (C-5), 151.5 (C-3), 195.8 (C-1).

### 2.4. High-Performance Liquid Chromatography (HPLC)

HPLC analysis was performed on a Waters 2695 series HPLC instrument equipped with a sample injector and a photodiode array UV/Vis detector (PDA) (Waters, United States). For all HPLC analysis, a CAPCELL PAK C18 UG120 (4.6 mm × 250 mm; 5 *μ*m, SHISEIDO Co., Japan) column was used as the stationary phase. Samples were prepared that contain 5 mg/mL concentration of AEM and 0.1 mg/mL concentration of (2*E*,5*E*)-6-hydroxy-2,6-dimethylhepta-2,4-dienal. Injection volumes for AEM and (2*E*,5*E*)-6-hydroxy-2,6-dimethylhepta-2,4-dienal were 50 uL and 10 uL, respectively. The mobile phase was composed of water (containing 0.1% formic acid) (A) and acetonitrile (B), with a gradient elution method: 0–5 min, 10% B; 5–15 min, a linear gradient from 10% B to 20% B; 15–60 min, a linear gradient from 20% B to 30% B; 60–80 min, a linear gradient from 30% B to 100% B; 80–90 min held at 100% B. Flow rate was 0.7 mL/min, and the peaks were detected at 270 nm. For coinjection analysis, 5 uL of (2*E*,5*E*)-6-hydroxy-2,6-dimethylhepta-2,4-dienal (0.1 mg/mL) was coinjected with 50 uL of AEM (5 mg/mL).

### 2.5. Animals

Six-week-old male ICR mice weighing 25 to 30 g were purchased from Orient Co. Ltd., Republic of Korea. Mice were housed six per cage and were maintained in temperature 20 ± 3°C under a 12/12 hr. light/dark cycle and adapted for 1 week before proceeding with treatment. Commercial pellet feed and water were allowed* ad libitum*. Animal handling and all the animal experiments were performed strictly adhering to the ethical guidelines of Institutional Animal Care and Use Committee at the Wonkwang University, Republic of Korea.

### 2.6. Y-Maze Test

The Y-maze is black, polyvinyl plastic maze consisting of three identical arms (40 cm × 3 cm × 12 cm). Spontaneous alternation [23] was tested as described previously [24]. In brief, mice were orally administered with AEM (62.5, 125, and 250 mg/kg) or tacrine (10 mg/kg) and injected with scopolamine (1 mg/kg, i.p.) or vehicle after 30 min. Each mouse was placed in the center of the Y-maze 30 min later and was allowed to explore freely through the maze during an 8 min session. The sequence and total number of arms entered were recorded as described before [25]. An entry is counted to have occurred when all four limbs are within the arm. The spontaneous alternation score (%) for each mouse is defined as the ratio of actual number of alternations to the possible alternation number (total number of entries – 2) multiplied by 100. The total number of entries into arms was assessed as a parameter representing locomotor activity [26, 27].

### 2.7. Passive Avoidance Task

The test was performed according to the method previously described [28]. In brief, assessment of acquisition and retention of the passive avoidance task were carried out using identical light and dark compartment (20 cm × 20 cm × 20 cm) (Jeungdo Bio and Plant Co. Ltd.) with an electrifiable grid floor separated by an entrance (5 cm × 5 cm) shutter. Mice underwent an acquisition trial and a retention trial 24 hours afterward. For the acquisition trial, the mouse was initially placed in the light compartment. After an acclimatization period of 10 s, the shutter was opened and it was closed after complete entry of the mouse into the dark compartment and an electrical foot shock (0.5 mA, 3 s) was delivered through the grid floor. Then mice were returned to their home cage. One hour before the acquisition trial, mice were administered with myrrh extracts (62.5, 125, and 250 mg/kg, p.o.), tacrine (10 mg/kg), or saline. After 30 min, scopolamine (1 mg/kg, i.p.) or vehicle was injected to induce memory impairment. After 24 h of acquisition trial, the mice were again placed in the light compartment for the retention trial and the latency time to enter the dark compartment was recorded and described as step through latency. The retention trial was set a limit of 300 s as cut-off time.

### 2.8. Western Blot Analysis

At the end of Y-maze tests, the mice were sacrificed by cervical dislocation and hippocampus was isolated, dissected, and homogenized in RIPA buffer (150 mM NaCl, 1% Triton X-100, 1% sodium deoxycholate, 0.1% SDS, 50 mM Tris-HCL, and 2 mM EDTA) supplemented with a protease and phosphatase inhibitor cocktail (Roche). Protein concentrations were determined using BCA assay kit (Thermo Scientific). Equal quantities of the protein (15–30 *μ*g) were subjected to SDS-PAGE in 10% gels and transferred to polyvinylidene difluoride (PVDF) membranes (Millipore). The blots were then incubated with antibodies specific for Akt, phospho-Akt (S473), ERK1/2, and phospho-ERK1/2 (T202/Y204) (Cell Signaling) (dilution 1 : 1000), followed by the corresponding secondary antibodies and finally developed using chemiluminescent reagents (Thermo Scientific). The relative intensities of specific protein bands were determined by densitometric scanning of images using ImageJ computer-assisted image analysis system.

### 2.9. Immunohistochemistry

The mice were anesthetized using 20% urethane (1 g/kg, i.p.) and perfused with 0.9% saline followed by 4% paraformaldehyde in 0.1 M phosphate buffer (pH 7.4). The brains were removed, postfixed in 4% paraformaldehyde, and dehydrated with 30% sucrose solution. Coronal sections (30 *μ*m) of mice brain were made through the hippocampus with a cryostat microtome and then immunostained using a R.T.U. Elite ABC kit (Vector laboratories). In brief, the sections were incubated in 0.3% hydrogen peroxide (H_2_O_2_) and washed in PBS. After 1 h incubation with blocking serum (2.5% horse serum), the sections were incubated with antibody directed against phosphorylated ERK, followed by biotinylated secondary antibody and then avidin-biotin-peroxidase complex mixture. The antigen-antibody complex was visualized by DAB chromogen (ImmPACT DAB Peroxidase Substrate Kit, Vector Laboratories). The brain sections were mounted, air-dried, dehydrated, cover slipped, and observed under a light microscope (Nikon, TS100). Photomicrographs were taken from hippocampus CA3 regions at ×20 magnification.

### 2.10. Statistics

All data are expressed as the mean ± SEM and the presented figures are representative of the series of experiments. Statistical significance of differences between test conditions were determined using one-way analysis of variance (one-way ANOVA) with Tukey's post hoc test for comparing multiple sets of data. A value of *p* < 0.05 was considered as significant.

## 3. Results

### 3.1. HPLC Analysis of AEM and Identification of a Major Component

**Figure 1:**
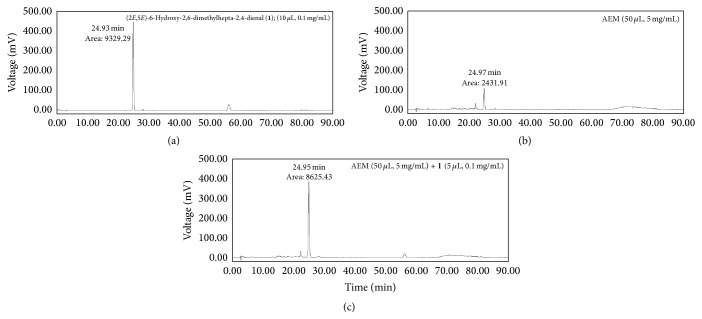
HPLC chromatograms of (a) (2*E*,5*E*)-6-hydroxy-2,6-dimethylhepta-2,4-dienal, (b) AEM, and (c) AEM coinjected with (2*E*,5*E*)-6-hydroxy-2,6-dimethylhepta-2,4-dienal. HPLC analysis was performed as described in Materials and Methods.

HPLC analysis of AEM obtained from the above elution method provided a major peak at 24.97 min (Figure 1(a)), which matched the retention time of (2*E*, 5*E*)-6-hydroxy-2,6-dimethylhepta-2,4-dienal (**1**). To further confirm the presence of** 1** in AEM, the marker compound** 1** was coinjected with AEM, and the resulting HPLC chromatogram (Figure 1(c)) showed that the area of the major peak has been increased without changes in retention time, peak width, and peak shape compared to the HPLC chromatogram of AEM. Therefore, the major component in AEM was assigned as (2*E*,5*E*)-6-hydroxy-2,6-dimethylhepta-2,4-dienal based on retention time matching and coinjection analysis.

### 3.2. Effect of AEM on Scopolamine-Induced Memory Impairment in the Y-Maze Test

**Figure 2:**
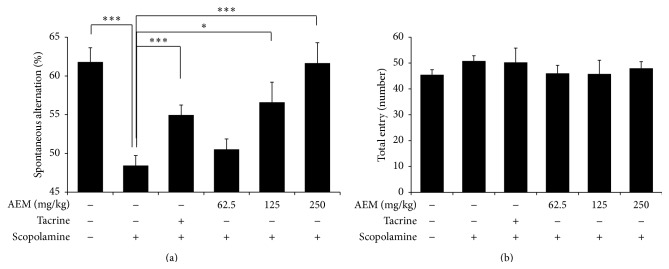
Effect of AEM on scopolamine-induced memory impairments in the Y-maze test. The mice under different groups were administered with equivalent volume of saline, tacrine (10 mg/kg, p.o.), or AEM (62.5, 125, and 250 mg/kg, p.o.) for seven days. Scopolamine (1 mg/kg, i.p.) was given to all the groups except control group 30 min before trial. The spontaneous alternation score (a) and numbers of arm entries (b) were recorded. Data are represented as mean ± SEM (*n* = 6 ~ 9) and the results are considered to be statistically significant at ^*∗*^ *p* < 0.05 and ^*∗∗∗*^ *p* < 0.001.

We first evaluated the effects of AEM on short-term memory function using Y-maze task. As shown in Figure 2(a), scopolamine (1 mg/kg, i.p.) significantly decreased the percentage of spontaneous alternation. The scopolamine-induced reduction of spontaneous alternation was significantly restored by the treatment with AEM (125 and 250 mg/kg) in a dose-dependent manner, suggesting the improved memory. The average spontaneous alternation of 125 and 250 mg/kg AEM was higher than that of 10 mg/kg tacrine. The total number of arm entries between the groups was not different suggesting that locomotion activity was not affected by scopolamine, tacrine, or AEM treatment (Figure 2(b)).

### 3.3. Effect of AEM on Scopolamine-Induced Memory Impairment in the Passive Avoidance Task

**Figure 3:**
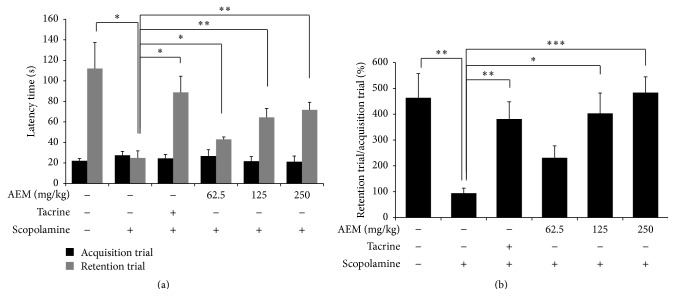
Effect of AEM on scopolamine-induced memory impairments in the passive avoidance task. The mice under different groups were administered with equivalent volume of saline, tacrine (10 mg/kg, p.o.), or AEM (62.5, 125, and 250 mg/kg, p.o.) for six days. Scopolamine (1 mg/kg i.p.) was given to all the groups except control group 30 min before acquisition trial. At 24 h after acquisition trial, a retention trial was performed 1 h after oral administration of saline, tacrine, or AEM. Latency time in the acquisition trial and retention trial (a) was recorded and the percentage ratio of retention trial to acquisition trial in each mouse (b) was calculated. Data are represented as mean ± SEM (*n* = 7 ~ 9) and the results are considered to be statistically significant at ^*∗*^ *p* < 0.05, ^*∗∗*^ *p* < 0.01, and ^*∗∗∗*^ *p* < 0.001.

Passive avoidance task was performed for testing the effect of AEM on scopolamine-induced memory impairment. As shown in Figure 3(a), the latency was not different between any of the groups during the acquisition trial. In the retention trial, the latency time of the scopolamine-treated group for entering the dark compartment was significantly shorter than the control group, indicating memory impairment. The latency of retention trial reduced by scopolamine treatment was ameliorated with treatment of AEM (62.5, 125, and 250 mg/kg) in a dose-dependent manner. In the percentage ratio of retention trial to acquisition trial, AEM treatment significantly reversed the scopolamine-induced reduction of latency time (Figure 3(b)).

### 3.4. Effect of AEM on the Phosphorylation of Akt and ERK1/2 in the Hippocampus

**Figure 4:**
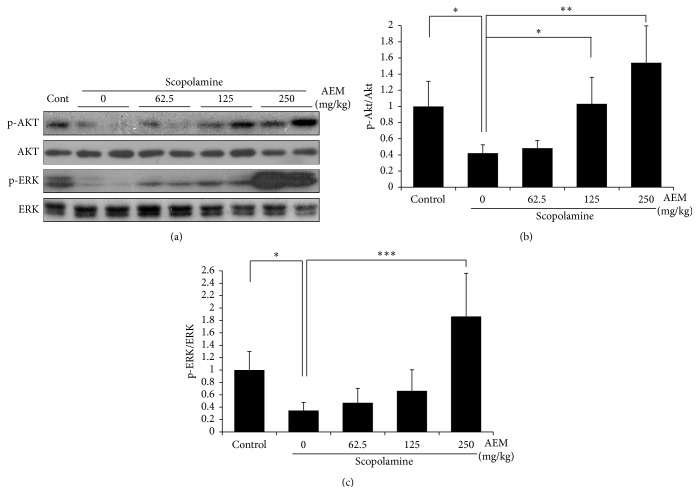
Effect of AEM on Akt and ERK phosphorylation in brain hippocampus. The hippocampus dissected from the randomly selected mice under different groups was used for Western blot analysis. The protein levels of phosphorylated Akt, total Akt, phosphorylated ERK, and total ERK were detected using specific antibodies. The representative blot (a) and the graph of quantification (b and c) were shown (*n* = 3). Quantifications were performed using densitometry. The results were normalized to total Akt or total ERK and expressed relative to the phospho-Akt or phosphor-ERK level of scopolamine-treated group. Each bar shown is the mean fold increase above control ± SEM and the results are considered to be statistically significant at ^*∗*^ *p* < 0.05, ^*∗∗*^ *p* < 0.01, and ^*∗∗∗*^ *p* < 0.001.

**Figure 5:**
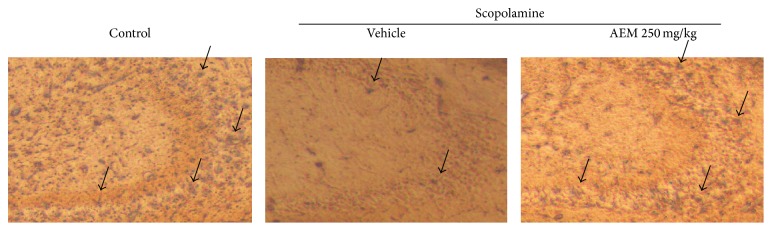
Effect of AEM on the phosphorylation of ERK in the brain CA3 region. The mice from each group were perfused 30 min after scopolamine injection. The brain sections were immunostained with antibody specific for p-ERK using the Elite ABC kit and then visualized with DAB chromogen. The black arrows indicate p-ERK antigen-antibody complexes. The representative images are shown (*n* = 4).

To elucidate the molecular mechanisms underlying the memory enhancing effect of AEM, we have examined the phosphorylation of Akt and ERK1/2 in the lysates of mice hippocampus. Western blot analysis clearly showed that scopolamine decreased Akt phosphorylation at serine 473 site and ERK1/2 phosphorylation at threonine 202 and tyrosine 204 sites. AEM (125 and 250 mg/kg) significantly reversed the scopolamine-suppressed phosphorylation of Akt and AEM (250 mg/kg) recovered the phosphorylation of ERK1/2 (Figure 4). In the Immunohistochemistry, scopolamine treatment decreased the immunoreactivity for the phosphorylated ERK in the hippocampal CA3 region (Figure 5). This reduction was recovered by AEM administration.

## 4. Discussion

In the present study, we demonstrate for the first time that the treatment of AEM ameliorates scopolamine-induced memory impairments. This effect was observed in the passive avoidance task and Y-maze test in mice. Furthermore, we found that AEM reversed the scopolamine-decreased phosphorylation of both Akt and ERK in the hippocampus of mice brains.

First, we estimated AEM with (2*E*,5*E*)-6-hydroxy-2,6-dimethylhepta-2,4-dienal as a representative constituent by HPLC. (2*E*,5*E*)-6-Hydroxy-2,6-dimethylhepta-2,4-dienal has been reported to be isolated from* Erechtites hieracifolia* [22],* Alpinia oxyphylla* [29], and Labdanum oil [30], but this compound was revealed as a component of myrrh for the first time in this paper.

The reduction of spontaneous alternation in the Y-maze test is known to represent short-term memory impairment in rodents [31]. In addition, retention latency in the passive avoidance task is considered to show the formation of long-term memory [32]. In the retention trial the latency time taken for mice to move into the dark compartment is increased owing to the knowledge of electric foot shock a day beforehand. In this study, the oral administration of AEM (125 and 250 mg/kg) increased spontaneous alternation more than that of 10 mg/kg of tacrine in Y-maze test (Figure 2(a)), suggesting its great efficacy in short-term memory deficit. In passive avoidance task, AEM treatment significantly increased the latency time in the retention trial in a dose-dependent manner, although the latency time in AEM groups was less restored than that in tacrine group (Figure 3). These results implicated the potential of AEM as a palliative in AD.

Several medications for AD treatment such as donepezil and rivastigmine caused some side effects such as hepatotoxicity, nausea, and diarrhea. Therefore, it is important to find relatively safe agents with few or no side effects. For the safety evaluation, AEM was administered at dose level of 200 mg/kg daily for 7 days in SD rats. Mortality, clinical signs, changes in body weight, hematology (WBC, RBC, Hgb, Hct, and PLT), serum chemistry (ALT, AST, PT, and APTT), gross observation, and organ weights were monitored in accordance with OECD guidelines. AEM did not produce treatment related signs of toxicity or mortality in any of the animals tested during the observation period (see Supplemental information in Supplementary Material available online at http://dx.doi.org/10.1155/2015/925432). Therefore, no observed adverse effect levels (NOAEL) were established for 200 mg/kg AEM in rats under the conditions of this study.

One of the important findings in this study is to show that AEM increased ERK and Akt activation in brain hippocampus. Western blot analysis (Figure 4) showed that AEM increased Akt and ERK phosphorylation in the hippocampus, compared to the scopolamine group. Furthermore, Immunohistochemistry (Figure 5) clearly showed that scopolamine treatment decreased ERK phosphorylation and AEM restored the reduction of ERK phosphorylation in mice hippocampus. Scopolamine inhibits the cholinergic neurotransmission through blocking mAChR. The activation of mAChR in the neurons leads to the elevation of intracellular calcium level, phosphoinositol turnover that activates ERK [9]. The activation of ERK1/2 is necessary for the establishment of long-term potentiation (LTP), which is associated with neuronal plasticity and development of memory [33]. It was reported that NMDA treatment or HFS-induced LTP increased ERK phosphorylation and PD098059, a MEK inhibitor, attenuated the induction of LTP in hippocampal CA1 area [34]. ERK is involved in the LTP-dependent transcriptional regulation through activation of transcriptional factor CREB [10, 35] in hippocampal dentate gyrus. Furthermore, ERK plays a crucial role for the induction of translation via phosphorylation of translation factors eIF4E, 4EBP1, and ribosomal protein S6 in the late LTP phase [36]. In addition, the activation of mAChR has been shown to induce Akt phosphorylation and thereby the inhibition of apoptosis in diverse cell types including neurons [37]. The potentiated action of acetylcholine through the injection of AChE inhibitors was reported to increase acute Akt phosphorylation in mice hippocampus [38]. Accordingly, AEM-induced activation of Akt and ERK in the hippocampus could be one of the molecular mechanisms underlying the memory enhancing effect of AEM.

In accordance with our findings, a wide range of natural product extracts, phytochemicals, and synthetic compounds have been reported to enhance memory in scopolamine-induced impairments through ERK and/or Akt activation. Stigmasterol, phytosterol present in foods, or *α*-amyrin and *β*-amyrin isolated from* Angelica keiskei* have been found to recover scopolamine-induced memory impairments in mice through enhanced ERK signaling in the hippocampus [28, 39]. Similarly, it has been reported that the scopolamine treatment decreased the phosphorylation of Akt and ERK and agmatine or honokiol reversed scopolamine-induced reduction of phosphorylated Akt and ERK in the brain and ameliorated memory impairments [14, 15, 40].

There have been few studies showing the effect of myrrh in the neuronal cells and the brain. The sesquiterpenes isolated from the resins of* C. myrrha* were reported to show neuroprotective effects against MPP^+^ induced neuronal cell death in SH-SY5Y cells [21]. In addition, we previously reported that 1*β*, 6*α*-dihydroxyeudesm-4(15)-ene, a sesquiterpene isolated from AEM, blocked lipopolysaccharide-induced inflammation by inhibiting the production of nitric oxide and PGE2 and by suppressing the protein expression of inducible nitric oxide synthase and cyclooxygenase-2 in BV2 microglial cell [41]. Although it has not been reported yet that myrrh has memory enhancing effects,* C. wightii, *another plant of the genus* Commiphora, *improved scopolamine- and streptozotocin-induced memory deficits [42].* C. wightii* treatment also caused the reduction of AChE activity and increment of GSH levels in the mice brains. Taken together, myrrh might have the potential benefits to alleviate various neurodegenerative diseases. To clarify this issue, further* in vivo* and* in vitro* studies will be needed.

## 5. Conclusion

Our study showed for the first time that AEM reverses the scopolamine-induced memory impairments in mice using the passive avoidance task and Y-maze test. Furthermore, AEM treatment upregulated Akt and ERK phosphorylation in the hippocampus of mice brain, suggesting that the memory improving effects of AEM treatment might be mediated at least partially through Akt and ERK activation. On the basis of our results, AEM is likely to be registered as a new promising candidate for the treatment of memory impairments.

## Supplementary Material

The safety evaluation of AEM in body weight, organ weights, hematology and serum chemistry showed neither toxicity nor mortality in SD rats administrated with 200 mg/kg of AEM daily for 7 days.
