# Supplementary material for: The Ameliorating Effect of Myrrh on Scopolamine-Induced Memory Impairments in Mice
Source: Evid Based Complement Alternat Med. 2015 Nov 9;2015:925432. doi: 10.1155/2015/925432 (PMC4655272; doi:10.1155/2015/925432)
Supplement: Supplementary file 1 — The safety evaluation of AEM in body weight, organ weights, hematology and serum chemistry showed neither toxicity nor mortality in SD rats administrated with 200 mg/kg of AEM daily for 7 days. [file 925432.f1.docx]

**Supplemental Information**


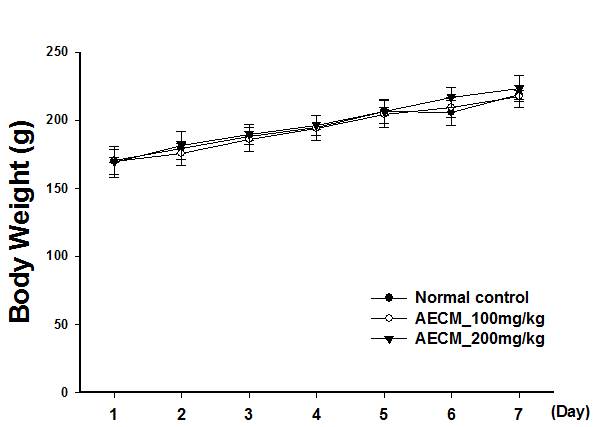


**Figure 1. Body weights in rats treated with AECM for 7days.** The values expressed as mean±S.D.

**Table 1. Absolute organ weights in rats treated with AECM for 7days.**

| **Organ (g)/**  **Dose(mg/kg)** | **AECM** | | |
| --- | --- | --- | --- |
|  | **0** | **100** | **200** |
| **Liver** | 10.9±0.63 | 11.1±1.27 | 9.90±0.63 |
| **Spleen** | 0.72±0.10 | 0.68±0.10 | 0.78±0.11 |
| **Stomach** | 1.32±0.10 | 1.37±0.17 | 1.35±0.06 |
| **Lung** | 1.41±0.07 | 1.47±0.13 | 1.58±0.12 |
| **Heart** | 0.98±0.06 | 1.01±0.07 | 0.99±0.04 |
| **Kidney** | 0.96±0.08 | 0.91±0.06 | 0.98±0.08 |
| **Testis** | 1.15±0.13 | 0.98±0.42 | 1.17±0.05 |
| **Brain** | 1.82±0.04 | 1.89±0.07 | 1.79±0.28 |

The values expressed as mean±S.D.

**Table 2. Relative organ weights in rats treated with AECM for 7days.**

| **Organ (%)/**  **Dose(mg/kg)** | **AECM** | | |
| --- | --- | --- | --- |
|  | **0** | **100** | **200** |
| **Liver** | 4.72±0.27 | 4.80±0.55 | 4.31±0.28 |
| **Spleen** | 0.31±0.04 | 0.29±0.04 | 0.34±0.05 |
| **Stomach** | 0.57±0.04 | 0.59±0.07 | 0.59±0.03 |
| **Lung** | 0.61±0.03 | 0.63±0.06 | 0.69±0.05 |
| **Heart** | 0.42±0.03 | 0.43±0.03 | 0.43±0.02 |
| **Kidney** | 0.41±0.03 | 0.39±0.03 | 0.43±0.03 |
| **Testis** | 0.49±0.06 | 0.42±0.18 | 0.51±0.02 |
| **Brain** | 0.78±0.02 | 0.81±0.03 | 0.74±0.12 |

The values expressed as mean±S.D.

**Table 3. Hematological parameters in rats treated with AECM for 7days.**

| **Parameters/**  **Dose(mg/kg)** | **AECM** | | |
| --- | --- | --- | --- |
|  | **0** | **100** | **200** |
| WBC (10^3^/μL) | 7.20±1.72 | 6.42± 1.53 | 8.52±2.45 |
| RBC (10^6^/μL) | 5.23±0.46 | 5.68±0.11 | 5.62±0.33 |
| Hgb (g/dL) | 12.1±0.85 | 11.4±0.15 | 11.6±0.19 |
| Hct (%) | 34.3±3.27 | 38.9±1.91 | 37.4±2.80 |
| PLT (10^3^/μL) | 856.4±36.9 | 932.6±24.1 | 1013.8±98.1 |

The values expressed as mean±S.D.

**Table 4. Clinical biochemical parameters in rats treated with AECM for 7days.**

| **Parameters /**  **Dose(mg/kg)** | **AECM** | | |
| --- | --- | --- | --- |
|  | **0** | **100** | **200** |
| AST (U/L) | 123.4±29.6 | 117.4±28.4 | 114.0±18.5 |
| ALT (U/L) | 40.8±4.1 | 40.6±4.8 | 44.6±6.1 |
| ALP(U/L) | 441.0±42.1 | 469.6±59.7 | 437.2±95.8 |
| Cholesterol (mmol/L) | 85.6±4.2 | 89.8±3.7 | 86.0±8.5 |
| Creatinine (μmol/L) | 0.2±0.0 | 0.2±0.0 | 0.2±0.0 |
| T.Bil (μmol/L) | 0.10±0.0 | 0.10±0.0 | 0.10±0.0 |
| BUN (mmol/L) | 16.2±1.2 | 16.0±2.9 | 15.5±1.2 |
| Glucose (mmol/L) | 190.4±27.2 | 194.8±11.4 | 189.4±14.6 |
| Protein (mmol/L) | 5.6±0.2 | 5.6±0.2 | 5.5±0.2 |
| PT (sec) | 15.9±0.7 | 16.6±2.8 | 16.0±0.7 |
| APTT (sec) | 21.9±1.7 | 22.0±5.1 | 22.0±0.4 |
| Na (mmol/L) | 141.0±0.7 | 141.0±1.4 | 139.2±0.8 |
| K (mmol/L) | 5.3±0.6 | 5.1±0.4 | 5.2±0.5 |
| Cl (mmol/L) | 102.8±2.2 | 102.8±1.6 | 102.2±0.4 |

The values expressed as mean±S.D.
